# Determinants of vector-borne avian pathogen occurrence in a mosaic of habitat fragmentation in California

**DOI:** 10.1186/s13071-025-06742-x

**Published:** 2025-03-15

**Authors:** Wilmer Amaya-Mejia, Lucas Pavan, Marie Lilly, Andrea Swei, Rodolfo Dirzo, Ravinder N. M. Sehgal

**Affiliations:** 1 University of California, Los Angeles, California USA; 2 Stanford University, Stanford, California USA; 3 Columbia University, New York, New York USA; 4 San Francisco State University, San Francisco, California USA

**Keywords:** *Haemoproteus*, *Borrelia burgdorferi*, Avian ecology, Habitat fragmentation

## Abstract

**Background:**

As habitat fragmentation increases, ecological processes, including patterns of vector-borne pathogen prevalence, will likely be disrupted, but ongoing investigations are necessary to examine this relationship. Here, we report the differences in the prevalence of Lyme disease (*Borrelia burgdorferi* sensu lato, s.l.) and haemoproteosis (*Haemoproteus* spp.) pathogens in avian populations of a fragmented habitat. *B. burgdorferi* s.l. is a generalist pathogen that is transmitted by *Ixodes pacificus* vectors in California, and *Haemoproteus* is an avian parasite transmitted by *Culicoides* vectors.

**Methods:**

To determine whether biotic (avian and mammalian abundance) or abiotic characteristics (patch size and water availability) correlated with infection prevalence change, we screened 176 birds sampled across seven sites in oak woodland habitat in northern California.

**Results:**

While biotic factors correlated with an increase in both pathogens, infection prevalence of *Haemoproteus* spp. was only associated with individual-level traits, specifically foraging substrate and diet, and *B. burgdorferi* s.l. was associated with community-level characteristics, both total mammal and, specifically, rodent abundance. Proximity to water was the only abiotic factor found to be significant for both pathogens and reinforces the importance of water availability for transmission cycles. Larger patch sizes did not significantly affect infection prevalence of *Haemoproteus,* but did increase the prevalence of *B. burgdorferi.*

**Conclusions:**

These results highlight that while environmental factors (specifically habitat fragmentation) have a limited role in vector-borne pathogen prevalence, the indirect impact to biotic factors (community composition) can have consequences for both *Haemoproteus* and *B. burgdorferi* prevalence in birds. Given the pervasiveness of habitat fragmentation, our results are of broad significance.

**Graphical abstract:**

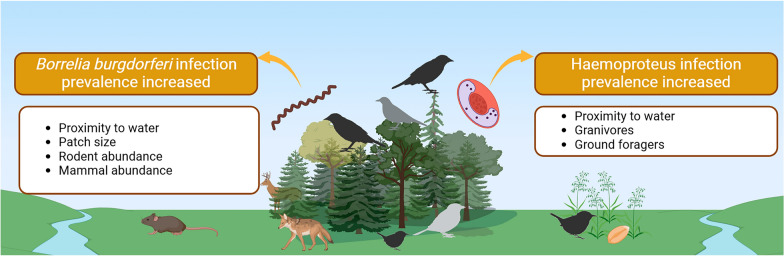

**Supplementary Information:**

The online version contains supplementary material available at 10.1186/s13071-025-06742-x.

## Background

Urban planning has led to increased habitat fragmentation, creating a mosaic of disturbance patch sizes, with impacts on ecosystem functions [1, 2]. For avian communities, these land-use changes have contributed to a global decline of biodiversity and a shift towards increasingly homogeneous communities, either through the increased presence of cosmopolitan, disturbance-associated species or a widespread distribution of select native species [3–8]. This means the impact of land-use changes can be particularly concerning for biodiversity hotspots, such as the California Floristic Province [9]. Despite these concerns, the loss of avian biodiversity is likely to continue as expanding urbanization results in greater habitat fragmentation [3, 10]. Of course, while this emphasizes the role of landscape reconfiguration on the spatiotemporal dynamics of a local population, there are other not-readily-evident factors that may have more subtle population regulatory roles.

Diseases represent one such subtle factor that can have a significant regulatory role in a community. This was most dramatically exemplified when naïve Hawaiian populations of birds were decimated following the introduction of avian malaria [11–13]. Our research aims to examine the effects of habitat fragmentation, and resulting landscape mosaic, on avian disease prevalence in a global biodiversity hotspot. Through this work, we hope to contribute to our understanding of the potential risks encountered by avian communities in our increasingly human-dominated landscapes.

To effectively monitor the avian disease ecology of urban environments, it is necessary to understand small-scale habitat variations that reflect nuanced landscape heterogeneity. This relationship between habitat variation and disease ecology is directly studied through individual capture methods in tandem with blood sample analysis, but this method requires considerable effort and may pose ethical complications when studying endangered species. An alternative approach could be to focus on identifying and mapping infection likelihood on the basis of relevant ecological characteristics. Ecological characteristics include extrinsic abiotic variables (e.g., water availability and elevation) and biotic variables, including life histories, host susceptibility, disease transmissibility, and current pathogen distribution. Once identified, pathogen-specific ecological characteristics can be reliably used as a less invasive approach to assess pathogen communities and disease risks.

An increase in habitat fragmentation is expected to affect disease systems, including those caused by avian haemosporidians infections such as *Haemoproteus*, the causative agent of haemoproteosis, among other pathogens [14]. Avian haemosporidians can be more prevalent and result in greater severity of infections in fragmented, urban habitats compared with the prevalence and impact observed in larger, more rural environments [15, 16]. The few available studies exploring the relationship between avian haemosporidians, host community composition, and abiotic environmental conditions have found that greater diversity of birds and proximity to water are positively correlated with infection prevalence [17–20]. This relationship is likely due to the haemosporidian vectors, biting midges, and their need for water to complete their life cycles [14, 21, 22]. Therefore, as both community composition and presence of water can be affected by habitat fragmentation, we expect that there are also implications for prevalence of avian haemosporidian pathogens.

In contrast to haemosporidians, *B. burgdorferi* sensu lato (s.l.), the causative agent of Lyme disease, can involve complex interactions with a greater diversity of hosts [23]. Local host communities, encompassing avian, non-avian, and vector community composition, can contribute to the maintenance of *B. burgdorferi* s.l. infections [23–25]. In particular, the role of birds in the transmission and maintenance of *B. burgdorferi* s.l. is still relatively understudied [23]. The vectors responsible for transmitting *B. burgdorferi* s.l. in California are ticks (e.g., *Ixodes pacificus*). A generalist vector, *I. pacificus* is capable of feeding and transmitting *B. burgdorferi* s.l. infections to various hosts including mammals and birds [26]. Vectors and hosts are known to be affected by habitat disturbance [27–29], underscoring the importance of community composition for this pathogen within landscape mosaics. Ticks are also sensitive to desiccation, suggesting that water availability through associated humidity could be equally important for *B. burgdorferi* s.l. infections as it is for avian haemosporidians [30]. The degree to which habitat fragmentation, water availability, and overall community composition shape local *B. borrelia* s.l. prevalence in birds could provide valuable insight on an understudied disease that is prevalent in both mammals and birds [23–25, 31].

Here, we assess commonalities between two avian-associated pathogens with dissimilar life histories and vectors to determine if there are consistent habitat-mediated patterns of infection prevalence. We detected differences in host susceptibility and transmission likelihood between specialist and generalist pathogens across landscape characteristics in live bird captures. We were specifically interested in understanding whether differences in the community composition of birds and abundance of co-occurring mammal species, as well as landscape characteristics, including patch sizes and water availability, are related to the prevalence of the two pathogens. We hypothesized that (1) infection prevalence of host-specialist parasites in the genus *Haemoproteus* would be correlated with birds, but not mammals, and (2) water availability would be the only landscape variable expected to affect susceptibility of birds to *Haemoproteus* pathogens. For the generalist pathogen *B. burgdorferi* s.l., (3) infections would increase in response to the proportion of susceptible hosts, including mammalian and avian, and (4) in addition to water availability, larger habitat size would also affect the susceptibility of birds to *B. burgdorferi* s.l. Although we screened for additional pathogens, including *Plasmodium*, the infection prevalence was too low in our study sites for proper analysis. Our reported results highlight the potential for developing low-cost, fine-scale pathogen monitoring across fragmented environments while minimizing human–animal interactions. Although the exact health impact of *Haemoproteus* and *B. burgdorferi* s.l. on birds in California is understudied, general studies have reported host mortality following severe, acute infections as well as low, chronic infections increasing the potential for certain bird species to serve as reservoir hosts for both pathogens [14, 32–34]. This work is of broad significance, given the increasingly evident relationship between avian conservation and public health globally.

## Methods

### Study site

This study was conducted on seven sites across the San Francisco Bay Area of California, USA, which is part of the California Floristic Province global biodiversity hotspot [9]. This region has a high degree of urbanization and supports nearly eight million people, a number that has grown by 8% in the last decade (www.vitalsigns.mtc.ca.gov/). On the basis of the Public Review Draft Environmental Impact Report published by Plan Bay Area 2040, 28% of the Bay Area’s land cover is identified as protected open space. These designated parks differ in size but occur within a Mediterranean climate with cool, wet winters and warm, dry summers that experience unique microclimates [35]. At each site, we delineated three separate 10 m radius vegetation sampling plots. The center of each plot was selected from a randomized compass bearing and located 15 m from one of the ten mist nets that were used during this study to capture birds. Within each plot, we surveyed tree stem density (here a tree was defined as anything larger than 10 cm DBH), canopy cover percentage (measured using a spherical crown densitometer at the center point of each plot), and understory density (measured using a 2 m cover pole and defined as the percent cover of vegetation obscuring this pole from an observation point 5 m away at a randomized compass bearing). As these results showed, within site habitat was comparable, consistent with a previous report that found all sites were similar in both vegetation and climatic conditions [28]. Owing to these similarities, we only examined the larger-scale landscape characteristics of habitat fragmentation and proximity to water. Additional file 1: Table S1 lists all survey data.

**Fig. 1:**
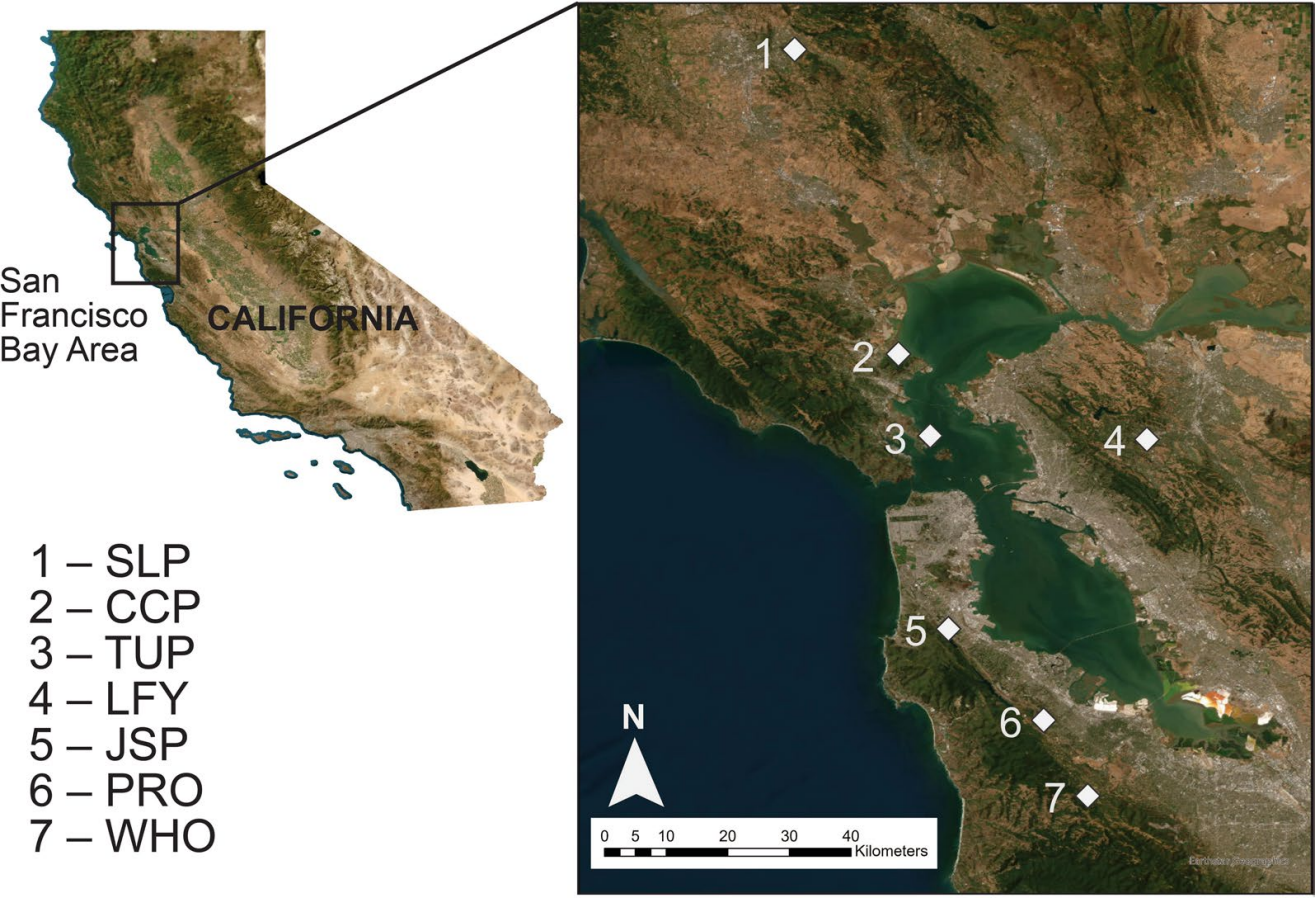
Site locations across northern California, USA. List of sites: Spring Lake Regional Park (SLP), China Camp State Park (CCP), Tiburon Uplands National Preserve (TUP), Lafayette Reservoir (LFY), Junipero Serra Park (JSP), Pulgas Ridge Open Space Preserve (PRO), and Windy Hill Open Space Preserve (WHO)

A total of seven study sites were selected because of their use in other long-term studies focused on the relationship between shifting mammal communities and *B. burgdorferi* s.l. distribution (Fig. 1). Previous studies on mammalian communities have been published for our study sites [28, 36], and our own previous research [24] provides details on the avian community as well. These sites include three located in San Mateo County, CA, USA [Junipero Serra County Park (JSP, 37.607917, −122.424111), Windy Hill Open Space Preserve (WHO, 37.36455, −122.22108), and Pulgas Ridge Preserve (PRO, 37.474749, −122.28512)]; two sites in Marin County, CA, USA (CCP, China Camp State Park (38.00843, −122.49703) and Tiburon Uplands Nature Preserve [TUP,37.889306, −122.450861)]; one site in Contra Costa County, CA, USA [Lafayette Reservoir Nature Area (LFY, 37.88394, −122.13472)]; and one site in Sonoma County, CA, USA [SLP, Spring Lake Regional Park (38.45225, −122.64833)]. Although some study sites are relatively close, our net placements exceeded the expected territory sizes and movement for the focal species (50–100 m) [37]. All sites are within an oak woodland habitat, and the dominant tree communities at all sites include *Quercus kelloggii* and *Quercus agrifolia*. The US Geological Survey (USGS) landcover database was used to assess habitat fragmentation, referring to the largest area within a site that is completely bounded by human infrastructure (https://www.usgs.gov/centers/eros/science/annual-national-land-cover-database). The National Water Dashboard was used to measure the distance to the nearest permanent water source on the basis of the Hydrologic Unit Code 12. Size and seasonal fluctuations were not accounted for within our approach.

### Field methods

Birds were captured during the breeding season at each site over two consecutive days between June and September in 2019 and 2020. Transects consisted of ten mist nets, each 12 × 2.5 m, opened at sunrise (06:00 h) for 5 h. This translated into 120 m of net coverage active for 20 h at each field site (680 m active for 80 h across the entire study). Birds were banded using appropriately sized federal aluminum bands. Morphometrics, body condition, bird taxonomy, age, and sex were determined on the basis of published characteristics [38]. Blood was collected from every bird via brachial venipuncture conducted with a 30 ga needle. Between 50 and 100 μL of blood were transferred via heparinized-microhematocrit capillary tubes into cryotubes containing 750 μL of lysis buffer [10 mM Tris pH 8.0, 100 mM etheylenediaminetetraacetic acid (EDTA), 2% sodium dodecyl sulfate (SDS)] for subsequent molecular analysis [39]. Two blood films were prepared for each bird and air-dried within 5–10 s after preparation. Slides were immediately fixed in methanol in the field and subsequently stained using 1:10 Giemsa: phosphate buffer (0.005 M KH_2_PO_4_ and 0.007 M Na_2_HPO_4_) at pH 7.2 [14] within 30 days. Slides were examined, but the low parasitemia proved too challenging to obtain sufficient observations and limited our ability to describe the morphospecies of the two novel lineages of haemosporidians; therefore, results were not included in our study. However, slides are available for future use.

### Pathogen screening and identification

Genomic DNA was preserved in the lysis buffer and stored at room temperature prior to freezing at −80 °C for long-term storage. Blood extraction was performed using Wizard SV Genomic DNA Purification System (Promega Corporation, Madison, WI, USA) following the manufacturer’s protocol. All subsequent polymerase chain reactions (PCRs) were conducted using GoTaq Flexi DNA Polymerase (Promega Corporation, Madison, WI, USA). DNA extraction quality was assessed by initial PCR amplification of the avian brain-derived neurotrophic factor gene to ensure the presence of amplifiable DNA [40]. Following verification of DNA extraction, nested PCR was used to test for *Haemoproteus* or *Plasmodium* by targeting the cytochrome *b* gene of the parasite’s mitochondria [41]. A nested PCR was used to screen for *B. burgdorferi* s.l. by targeting the *B. burgdorferi* s.l.-specific 5S-23S rRNA intergenic spacer region [24, 42, 43]. Nested PCR products were submitted for Sanger sequencing at ElimBio (CA, USA). Sequences were used to identify the parasite genus by comparing against the MalAvi and NCBI BLAST databases. Any haemosporidian sequence from our study that differed from a published sequence by over 1% or 4 bp was considered novel. Additional phylogenetic analysis will be reported elsewhere. A subset of birds was screened for the presence of *Trypanosoma*, *Leucocytozoon*, or avian pox, but owing to low infection prevalence of each, these were excluded from subsequent analyses [39, 44, 45]. Confidence intervals (95%) for infection prevalences were calculated using the methods listed in ref. [46]. In short, the qbeta function was run using base R version 1.2.5033 (RStudio Team 2019) with the shape one parameter set to the number of positive samples + 1 and the shape two parameter set to total sample size—positive samples + 1.

### Statistical analysis

A series of independent models were conducted for each genus of pathogen. The infection prevalence of *Haemoproteus* and *B. burgdorferi* s.l. was set as the dependent variable in their respective sets of models. We applied binomial logistic regressions to infection data derived from avian blood samples using the package stats in R 4.1.2. To avoid overparameterization, each series consisted of three separate models (six models total—three per focal pathogen) comparing different potential determinants of infection prevalence. We only report on the best explanatory factors, but other variables including rodent abundances were also initially examined. The first was the site characteristic model (SCM) examining the effect of site identity, the area of contiguous habitat cover, and the distance to the nearest permanent water source (the latter two parameters determined using USGS land cover maps). The second model was the total community model (TCM), which included the abundance and diversity of rodents, non-rodent mammals, and birds at each site determined either from previously published data or from avian sampling conducted as part of this study. All surveys of mammal community metrics were completed using both live trapping (with Sherman traps) and camera trapping methods. The final model was the avian community model (ACM), which included the effects of bird sex, mass, and age, determined in the field, as well as diet guild, foraging guild, and nesting guild, which were categorized using data published by the Birds of the World Handbook (http://www.birdsoftheworld.org/) and related publications [47–49]. Models are outlined in Table 1. These three models were selected to approximate the effects of variance on host-specificity, driven by differences in avian community composition (the ACM), and transmissibility driven by differences in broader community composition (the TCM) or environmental characteristics (the SCM). Statistical significance of variables in each model was calculated using the likelihood-ratio test.

**Table 1:** Models of avian host infection prevalence as a response to abiotic and biotic factors

Model	Response variable	Parameter	LR *X*^2^	DF	*p-*Value
SCM					
	*B. burgdorferi* s.l.	Site	33.208	6	< 0.001***
		Site area	7.634	1	0.006**
		Distance to water	13.389	1	< 0.001***
	*Haemoproteus *spp.	Site	22.837	6	0.001**
		Site area	1.707	1	0.191
		Distance to water	5.104	1	0.024*
ACM					
	*B. burgdorferi* s.l.	Diet	1.651	2	0.438
		Foraging	0.519	3	0.915
		Nesting	3.379	4	0.497
		Sex	1.997	2	0.368
		Age	0.148	1	0.701
		Body condition	0.937	1	0.333
	*Haemoproteus *spp.	Diet	10.886	2	0.004**
		Foraging	9.329	3	0.025*
		Nesting	4.778	4	0.311
		Sex	6.398	2	0.041*
		Age	0.046	1	0.831
		Body condition	0.397	1	0.528
TCM					
	*B. burgdorferi* s.l.	Avian richness	0.954	1	0.329
		Rodent richness	8.504	1	0.004**
		Non-rodent richness	3.507	1	0.041*
	*Haemoproteus *spp.	Avian richness	0.314	1	0.575
		Rodent richness	0.294	1	0.588
		Non-rodent richness	1.488	1	0.223

*, **, ***Statistically significant (*p* < 0.05; < 0.01; < 0.001). *SCM* site characteristics model, *ACM* avian community model, *TCM* total community model,* LR X*^2^, likelihood ratio test, *DF* degrees of freedom

## Results

### Pathogen prevalence

In the 2019 and 2020 breeding seasons, 218 individuals, corresponding to 22 distinct species of birds, were captured using mist nets, and samples were successfully collected from 176 individuals and screened for parasitic infections. A detailed list of all samples is available (Additional file 1: Table S2). Parasite lineages were diverse, but because abundance was relatively low, infections will be discussed at the genus level unless otherwise stated. Using PCR-based detection methods, 18.2% (confidence intervals, CI 13.2–24.6%) of birds were found to have *Haemoproteus* infections, 1.7% (CI 0.6–4.9%) had *Leucocytozoon* infections, and 1.1% (CI 0.4–4.0%) had *Plasmodium* infections. A total of 14.8% (CI 10.3–20.8%) *B. burgdorferi* s.l. infections were detected. Only one dark-eyed junco (*Junco hyemalis*) from WHO was found to be infected with *Borrelia bissettiae*, corresponding to 0.6% (CI 0.1–3.1%) of all samples. The most abundant bird species, dark-eyed junco, had high prevalence of both *Haemoproteus* (30.4%, CI 21.3–41.3%) and *B. burgdorferi* s.l. (15.2%, CI 8.9–24.7%) infections, but sample sizes were still too low for species-specific analyses. Coinfections of *Haemoproteus* and *B. burgdorferi* s.l. were detected in 4.0% (CI 2.0–8%%) of samples. These coinfections include the single dark-eyed junco infected with *B. bissettiae* also being infected with *Haemoproteus* spp. JUNHYE3.

The majority of species studied were represented by fewer than five individuals. Therefore, our analysis focused on guilds (granivores, insectivores, or omnivores) rather than species. Infection prevalence of *Haemoproteus* was highest in granivores (29.7%, CI 21.3–39.8%) while the prevalence of *B. burgdorferi* s.l. was highest in omnivores (18.2%, CI 5.5–48.4%). *Plasmodium* infections were uncommon (omnivores: 0%, CI 0.2–26.5%; granivores: 1.1%, CI 0.3–5.9%; and insectivores: 1.4%, CI 0.3–7.2%). Specific details about infection prevalence per individual and across sites are included in Additional File 1: Table S2.

**Fig. 2:**
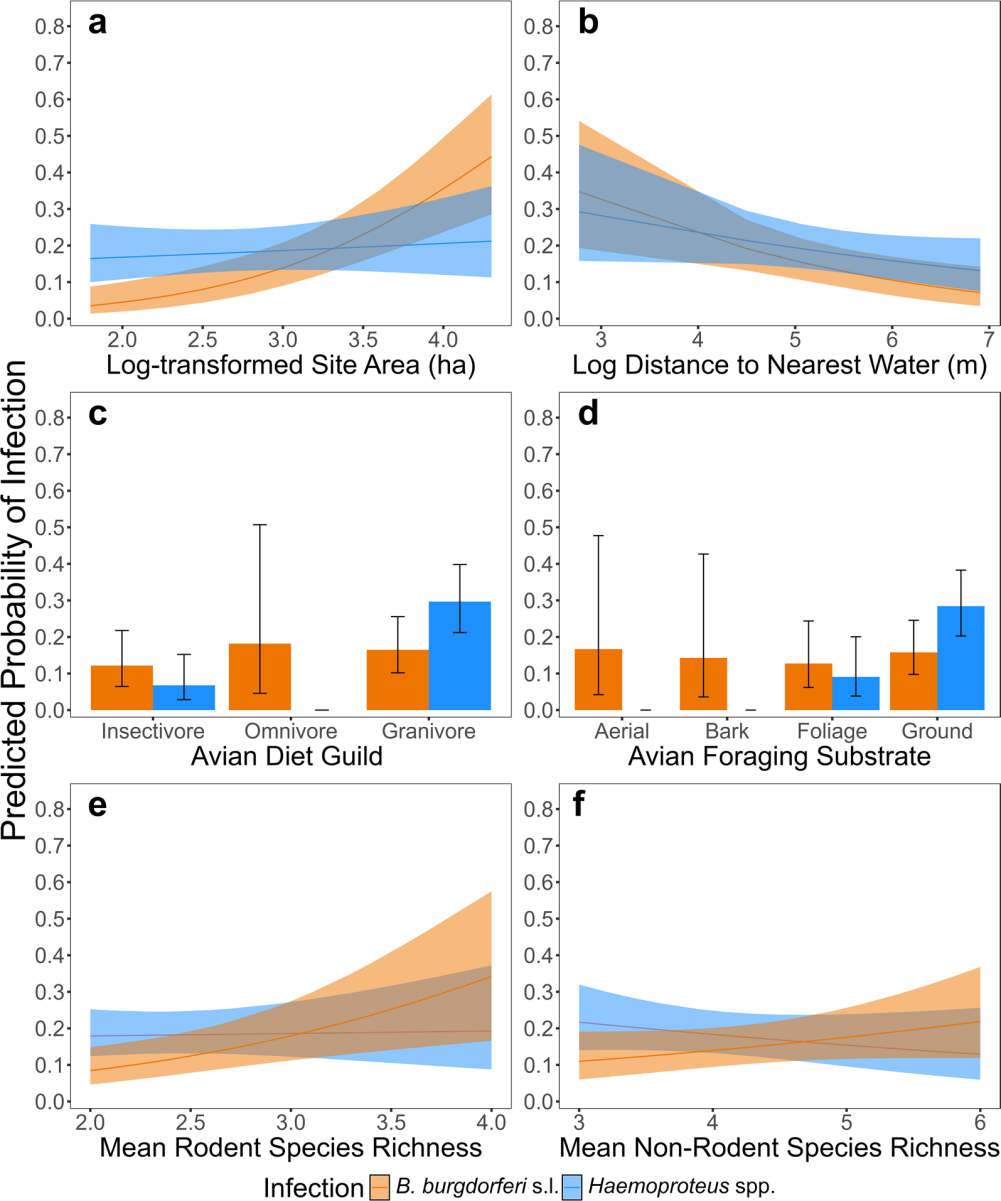
Plots of significant model predictors. Line plots show response of infection prevalence of *B. burgdorferi* s.l. (orange) and *Haemoproteus* (blue) to geographic and biotic environmental characteristics. SCM includes **a** habitat size and **b** proximity to fresh water. ACM includes **c** diet guild and **d** foraging substrate. TCM includes **e** diversity of non-rodent communities, and **f** diversity of rodent communities

Both pathogens showed a correlation with site characteristics (Table 1). *Borrelia burgdorferi* s.l. showed a response to all parameters including site identity, was positively correlated with habitat patch size, and negatively correlated with proximity to water (Fig. 2a and b), Similarly, *Haemoproteus* infection prevalence increased when in closer proximity to water (Fig. 2b) but was statistically independent of fragment size. Model coefficients are provided in Additional File 1: Table S3.

Total community composition did not change *Haemoproteus* infection prevalence, but *B. burgdorferi* s.l. infections were significantly correlated with mammal communities (Table 1, TCM). Both rodent species richness (Fig. 2e) and non-rodent mammal species richness (Fig. 2f) were positively correlated with *B. burgdorferi* s.l. infection prevalence. Avian community richness did not correlate with infection prevalence of either pathogen.

Only *Haemoproteus* infection prevalence was strongly affected by individual avian characteristics (Table 1, ACM). To determine individual susceptibility of the different bird species sampled, an initial model was run to determine if the probability of infection prevalence changed when considering sex, age, body condition, nesting substrate, foraging substrate, and primary diet composition. Of these, sex, diet (Fig. 2c), and foraging substrate (Fig. 2d) were significant and were shown to contribute to risk of *Haemoproteus* infections. *Borrelia burgdorferi* s.l. infection prevalence did not correspond with any of the examined host characteristics.

### Pathogen lineage diversity

Across all sites, there were a total of 13 unique lineages (determined by a difference > 4 bp in the cyt *b* gene sequence) of haemosporidian parasites. Eleven of these lineages have sequences previously recorded and are presented with previously listed lineage names and accession numbers (Additional file 1: Table S2). The remaining two lineages differ from recorded lineages by over 1% or 4 bp, suggesting these could represent novel lineages. These include *Haemoproteus spp. SPIPSA01* (GenBank accession number: PQ765730), found in a lesser goldfinch (*Spinus psaltria)* and *Haemoproteus spp.* *JUHYE26* (GenBank accession number: PQ765731), found in a dark-eyed junco. *B. burgdorferi* species in this study primarily consist of *B. burgdorferi *sensu stricto and one confirmed *B. bissettiae* infection detected. Four hosts were confirmed to be infected with *B. burgdorferi* s.l. but species identification could not be performed.

## Discussion

Disease prevalence is, in part, the result of how pathogens respond to environmental changes. In the case of vector-borne pathogens, infection prevalence should vary when pathogens demonstrate unique life histories. To this end, our study focused on determining what site characteristics are most likely to result in infection hotspots. We compared the prevalence of two unrelated vector-borne pathogens, *B. burgdorferi* s.l. and *Haemoproteus*, within the avian community in northern California. While some site characteristics correlated with differences in infection prevalence for both pathogens, community composition showed pathogen-specific variation.

For *Haemoproteus* spp., infections with these pathogens are transmitted within the avian hosts, *Culicoides* vector complex [50–52]. Given the relative specialization of the *Haemoproteus*-avian disease system, we expected to see infection prevalence respond to the avian host community composition. This was partially consistent with our results, as infection prevalence was higher in specific guilds and differed on the basis of diets (Fig. 2C, D). Ground foragers and granivorous species had the highest infection prevalence, likely the result of increased accessibility of these birds to the vectors compared with other guilds [53, 54]. However, it should be noted that avian species richness within a habitat was not predictive of *Haemoproteus* infection prevalence. This suggests that the ecological niche of a bird, rather than overall community composition, is more important for predicting whether birds become infected. This conclusion is consistent with results reported in other systems [17, 53, 55, 56].

The infection prevalence of *Haemoproteus* was positively correlated with proximity to water. This was consistent with our initial hypothesis, in which we expected areas closest to water would have the highest *Haemoproteus* infection prevalence. As water availability is necessary for the biting midge life cycle [21], we expected that proximity to water would increase vector abundance and subsequently increase *Haemoproteus* infection prevalence.

Environmental factors that affect host communities had a weaker impact on *Haemoproteus* infection prevalence. We did not find a connection between habitat size and *Haemoproteus* infection prevalence. This was expected as habitat size should impact host communities, specifically host species richness, not necessarily vector abundance [27]. When evaluating *Haemoproteus* lineages, many were previously reported in a study conducted in Alaska [57]. The significant environmental differences between California and Alaska highlight that the distribution of *Haemoproteus* pathogens is more likely regulated by host species rather than by broader environmental conditions. Collectively, our results suggest that *Haemoproteus* infection prevalence is regulated by host species and potentially vector communities rather than abiotic characteristics or community-level variations.

Our second pathogen of interest was *B. burgdorferi* s.l. This bacterial pathogen is locally transmitted by several ixodid ticks, notably *I. pacificus*, that feed on a wide range of blood meal hosts [26, 58]. With this large pool of susceptible hosts, we expected to observe a positive correlation between *B. burgdorferi* s.l. and host community composition. We did find that infection prevalence was positively correlated with mammalian species richness, for both rodent and non-rodent community richness. However, there was no correlation with avian community composition. The lack of correlation between avian community composition and *B. burgdorferi* s.l. supports the idea that birds have a relatively marginal role in Lyme disease transmission within this habitat [59]. Unlike mammals, and especially rodents, birds are not primary reservoirs of *B. burgdorferi* s.l.; therefore, even as bird communities composition changes, infection prevalence is expected to remain consistent [26, 60, 61].

When examining abiotic habitat characteristics, we expected infection prevalence of *B. burgdorferi* s.l. to increase in higher quality habitats. This hypothesis was supported, as we found that birds sampled from larger habitats and habitats near water had a higher prevalence of *B. burgdorferi* s.l. Although it is difficult to disentangle the effects of habitat disturbance from the effects on community composition, we can provide some insight on the basis of our results. We propose that the increase in *B. burgdorferi* s.l. infection prevalence in response to proximity to water is most likely related to the dependence of *I. pacificus* on specific humidity. However, a future study could determine if the presence of water correlates with behavioral changes in birds [62, 63] and whether this alters pathogen transmission. The role of habitat patch size is especially complex owing to its impacts on species diversity, abundance, and interactions [27, 28]. Of these, we could only examine diversity and abundance, and we found that species abundance, whether avian or mammalian, was not significantly correlated with *B. burgdorferi* s.l. infection prevalence in birds.

Although additional studies are necessary, previous literature suggests that increased biodiversity can have a nuanced role in maintaining *B. burgdorferi* s.l. within this system [36, 64]. One prevailing hypothesis known as the “dilution effect” posits that increased species diversity may decrease pathogen infection prevalence by increasing the proportion of noncompetent reservoir hosts and decreasing the proportion of highly competent reservoir hosts [65, 66]. Nevertheless, there is mixed empirical evidence across systems regarding the role of diversity in pathogen transmission [67]. While the original idea of the dilution effect was first proposed in the Lyme disease system in the northeastern USA [65, 68], other studies that involved highly fragmented landscapes in the eastern [69] and western USA [36] have found a positive relationship between increasing mammalian diversity and Lyme disease risk. This variability and the differing community assemblies in regional Lyme disease systems could explain the results found in our study. These results emphasize the complexity of studying Lyme disease and highlight the need for ongoing studies into this zoonotic pathogen.

Our study was based on a relatively small sample size that consisted of various bird species. To account for this limitation, we analyzed the data with a focus on one of three broad categories: site, bird characteristics, and community composition. However, many of the variables within these categories were highly correlated and could have obscured some of the mechanisms driving the patterns. Of these categories, only the result of our SCM was relatively robust, showing the same result when a negative binomial model was performed (SI Table 3). With future studies, it can be possible to verify and expand on these initial findings.

The lack of information on haemosporidian vectors is critical, but particularly challenging to obtain. Studies on the specific vectors in California that transmit *Haemoproteus* are sparse but based on broader studies, and the general life history of biting midges (genus: *Culicoides*) has suggested that water is necessary to maintain the larval stages and could subsequently be responsible for the increase in *Haemoproteus* infection prevalence [21, 51, 70–73]. Fully determining whether the correlation between water and *Haemoproteus* infection prevalence is the result of an increase in vector populations or due to increased concentration of hosts around a body of water requires further examination. In the case of *B. burgdorferi* s.l., abundance of infected nymphal ticks was found to correlate with infection prevalence, further emphasizing the importance of data on vectors for these disease systems [24]. Clearly, the role of vectors is an aspect that warrants further research.

An important distinction is that our study examines infection prevalence, but not transmission. While age was included as one of the factors in our model, the low parasitemia observed and low likelihood of obtaining wild birds during the acute infection stage [14] suggests that many birds were previously infected. This distinction is especially relevant for *Haemoproteus,* as the *B. burgdorferi* s.l. infections are likely locally acquired [24, 74]. A related concern is that birds are highly mobile, which can complicate determination of where infections are acquired. While connectivity between sites by movement was not directly assessed within our study, there were no instances of recapturing birds across study sites, but this does not discount the possibility of annual movement between sites. In the case of our most commonly captured bird, the dark-eyed junco, the distance between sites exceeded the projected home range sizes 7.97 ha during the breeding season [37]. Bird behaviors, such as feeding and general proximity to the ground, may play a role in infection probability. Additionally, while mobility could impact our results, these should result in increased homogenization that would have reduced our ability to detect differences between sites. Last, while our results captured the locations of bodies of water during the study, further research should examine possible effects of seasonal fluctuations in water availability.

## Conclusions

Human-mediated habitat changes are increasing and, as a result, we are observing downstream consequences that are altering ecological communities. Although these habitat changes are expected to increase the homogeneity of bird communities, landscape mosaics present intrinsic and extrinsic factors that inform prevalence patterns of other wildlife and vector populations. Such combined effects are expected to have cascading effects on the dynamics of avian diseases. For example, we determined that *Haemoproteus* was impacted by avian host communities and water availability, consistent with other studies. In addition, as one of the few studies exploring the relationship between local bird communities and *B. burgdorferi* s.l., we found that mammal communities and habitat quality are important for the maintenance of this pathogen. Additionally, the observed differences in predictors between pathogens highlights that blanket vector mediation strategies may not be sufficient to regulate unique pathogens. Whether these hosts and pathogens continue to maintain their current patterns or manage to evolve in response to habitat changes could prove vital for both human and ecological health, but by prioritizing regions that are likely to be hotspots, we can monitor and prepare for these changes.

## Supplementary Information

Additional file 1: Table S1. Site vegetation survey summary. Table S2. Detailed individual-level infection, morphological, and guild characteristics. Table S3. Detailed model coefficients and negative binomial regression output

## Data Availability

The datasets supporting the conclusions of this article are included within the article and its additional files.

## Abbreviations

*B. burgdorferi* s.l.: *Borrelia burgdorferi* sensu lato
SCM: Site characteristics model
ACM: Avian community model
TCM: Total community model
